# Study on a Detection Technique for Scholte Waves at the Seafloor

**DOI:** 10.3390/s22145344

**Published:** 2022-07-18

**Authors:** Minshuai Liang, Liang Wang, Gaokun Yu, Yun Ren, Linhui Peng

**Affiliations:** 1 Department of Marine Technology, Ocean University of China, Qingdao 266100, China; lms2423@stu.ouc.edu.cn (M.L.); gkyu@ouc.edu.cn (G.Y.); penglh@ouc.edu.cn (L.P.); 2 State Key Laboratory of Acoustics, Institute of Acoustics, Chinese Academy of Sciences, Beijing 100190, China; renyun@mail.ioa.ac.cn

**Keywords:** Scholte wave detection, multilayer elastic bottom, acoustic pressure field, source depth, propagation distance

## Abstract

Scholte waves at the seafloor have significant potential for underwater detection and communication, so a study about detecting Scholte waves is very meaningful in practice. In this paper, the detection of Scholte waves at the seafloor is researched theoretically and experimentally. Acoustic models with the multilayer elastic bottom are established according to the ocean environment, and a tank experiment is designed and carried out to detect Scholte waves. Different from detecting Scholte waves in the seismic wavefield, a technique for detecting Scholte waves in the sound pressure field is proposed in this paper. The experimental results show that the proposed technique can detect Scholte waves effectively, and there are no problems such as seabed coupling and the effect of wave speeds. Furthermore, the results also show that this detection technique is still effective in conditions with a sediment layer. The existence of sediment layers changes the acoustic field conditions and affects the excitation of Scholte waves.

## 1. Introduction

In marine settings, the waves trapped near the fluid–solid interface are called Scholte waves [1]. Scholte waves are a kind of interface wave, and they are expected to have a longer traveling path or less transmission loss than waves in water [2,3]. These characteristics make Scholte waves a great prospect in underwater detection and communication, leading to research interests in ocean acoustics. Currently, Scholte waves have been applied in many areas, such as geoacoustic inversion [4,5,6,7,8], acoustic source localization [9,10], and imaging [11]. Therefore, how to detect Scholte waves effectively has significant meaning in ocean detection.

In existing methods, the seismic field is often applied to detect Scholte waves. Seismic measurement equipment such as Ocean Bottom Seismometers (OBSs) [12,13,14] and geophones [6,9] is used to record the seismic signal at the ocean bottom. Then, the velocity characteristics in the fields can be extracted from the recorded signal [2,8]. Finally, Scholte waves are identified according to the velocity characteristics.

In the measurement process, the seismic measurement equipment mainly records the seismic wave signal on the seafloor through the coupling between the equipment and seafloor, so their measurements are susceptible to the bottom type and seafloor topography [15,16]. Compared with seismic equipment, the hydrophone is immune to these issues, making it more suitable for measuring underwater acoustic signals.

In the stage of signal processing, the velocity characteristics of Scholte waves are used to identify Scholte waves as mentioned above. The velocities of Scholte waves have two features. One is slower speed, and their speeds are slower than other waves in the acoustic field. A later arriving signal with high energy can be observed in the time series [9,12,17,18]. Another is that the velocities of Scholte waves are dispersive in the waveguide [12]. Investigators have proposed many practical techniques to extract the dispersion curve of Scholte waves, the most extensive of which is the approach based on domain transformation [5,7,8,19,20,21]. The extracted dispersion curve can be used to identify Scholte waves at the seafloor. These velocity-based approaches can identify Scholte waves effectively when the Scholte wave velocity differs largely from the acoustic waves in water. However, they will be invalid when the velocities are close to each other.

This paper introduces a new technique to detect Scholte waves based on acoustic pressure field measurement and researches the detection of Scholte waves theoretically and experimentally. Acoustic models with the multilayer elastic bottom are established. The Scholte wave is identified according to the excitation and propagation characteristics. Scaled-down tank experiments for detecting Scholte waves are designed and carried out. Firstly, the detection principle is introduced. The characteristics of Scholte waves that vary with the source depth and propagation distance are researched. Then, the scaled-down experiment is introduced in detail. Acoustic field features in the water tank are analyzed. Measurement and analysis results of this experiment are presented. Furthermore, the research in conditions with sediment layers is introduced. Finally, the summary and conclusions are presented.

## 2. Detection Principle

### 2.1. Acoustic Model

**Figure 1:**
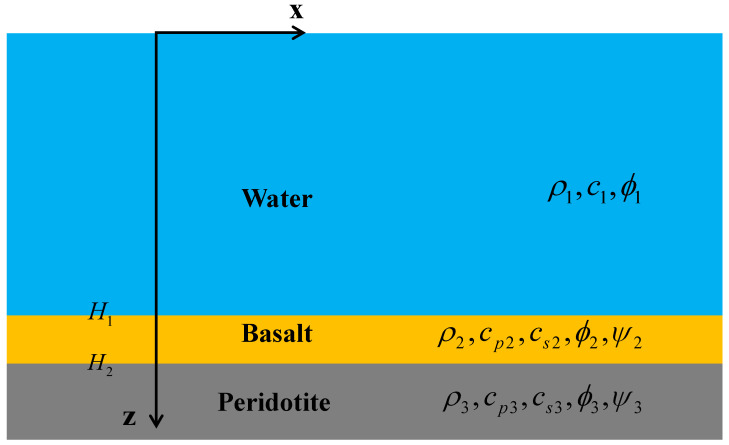
Schematic of the acoustic model for the marine environment. $H_{1}$ and $H_{2}$ are the interface depth for “water–basalt” and “basalt–peridotite”.

Ocean acoustic studies generally assume the ocean bottom as the fluid bottom [22], but this assumption is not suitable for the research in this paper. The elastic bottom plays an indispensable role in the excitation and propagation of Scholte waves, so the ocean bottom is an elastic medium with shear waves in this research. The marine environment is simplified into a three-layer model consisting of seawater, basalt, and peridotite, as shown in Figure 1, based on Hamilton’s studies [23] and the results of geological surveys [24].

The typical deep ocean environment is researched in this study. Referring to related research [25], the media parameters in the model are shown in Table 1. The KRAKENC program is used to analyze the acoustic field in this model. The phase velocities of the Scholte wave and normal modes at 10 Hz are calculated and listed in Table 2. The Scholte wave in the acoustic field is treated as the zeroth mode. These data show that the velocities of the Scholte wave and normal modes are extremely close, which means that the Scholte wave in the field cannot be identified using velocity features.

A new technique is proposed to detect Scholte waves according to the excitation and propagation characteristics.

### 2.2. Elastic Normal Modes

In order to interpret the detection principle, an elastic normal mode solution is presented for this range-independent model with the elastic bottom. Assume that all layers in the marine environment are isotropic media. Subscripts *i* = 1, 2, 3 are used to represent acoustic parameters in seawater, basalt, and peridotite, respectively. $\rho_{i}$ is density; $c_{1}$ is seawater sound velocity; $c_{p2}$ and $c_{p3}$ are compressional wave velocities (P wave); $c_{s2}$ and $c_{s3}$ are shear wave velocities (S wave). $H_{1}$ and $H_{2}$ represent the interface depths for “water–basalt” and “basalt–peridotite”.

The acoustic field is expressed by velocity potentials. $\phi_{1}$ is the potential in water; $\phi_{i}$ and $\Psi_{i}{\vec{e}}_{y}$ (i=2,3) are compressional and shear potentials in the elastic bottom, respectively. Potentials in the water and bottom satisfy the wave equations [26].

(1) $$\frac{\partial^{2}\phi_{1}}{\partial z^{2}}+\frac{\partial^{2}\phi_{1}}{\partial x^{2}}=\frac{1}{c_{1}^{2}}\frac{\partial^{2}\phi_{1}}{\partial t^{2}}$$

(2) $$\frac{\partial^{2}\phi_{i}}{\partial z^{2}}+\frac{\partial^{2}\phi_{i}}{\partial x^{2}}=\frac{1}{c_{pi}^{2}}\frac{\partial^{2}\phi_{i}}{\partial t^{2}}$$

(3) $$\frac{\partial^{2}\psi_{i}}{\partial z^{2}}+\frac{\partial^{2}\psi_{i}}{\partial x^{2}}=\frac{1}{c_{si}^{2}}\frac{\partial^{2}\psi_{i}}{\partial t^{2}}$$

A time–harmonic plane wave with the time dependence $e^{-i\omega t}$ is considered. Subsequently, all potentials can be represented as follows:

(4) $$\phi_{i}=Z_{i}\left(z\right)e^{i\left(kx-\omega t\right)}$$

(5) $$\psi_{i}=Z_{si}\left(z\right)e^{i\left(kx-\omega t\right)}$$

where ω=2πf is the angular frequency and k=ωωcc is the wavenumber. $Z_{i}\left(z\right)$ and $Z_{si}\left(z\right)$ are depth-dependent functions for compressional and shear waves; they satisfy the homogeneous depth-separated wave equations.

(6) $$\left[\frac{d^{2}}{dz^{2}}+\left(k_{i}^{2}-k^{2}\right)\right]Z_{i}\left(z\right)=0$$

(7) $$\left[\frac{d^{2}}{dz^{2}}+\left(k_{si}^{2}-k^{2}\right)\right]Z_{si}\left(z\right)=0$$

The general solutions for potentials can be obtained by the free surface condition and radiation condition at infinity.

(8) $$\phi_{1}=-2A\sinh\left(k\xi_{1}z\right)e^{i\left(kx-\omega t\right)}$$

(9) $$\phi_{2}=\left(B_{1}e^{-k\xi_{2}z}+B_{2}e^{k\xi_{2}z}\right)e^{i\left(kx-\omega t\right)}$$

(10) $$\psi_{2}=\left(C_{1}e^{-k\xi_{s2}z}+C_{2}e^{k\xi_{s2}z}\right)e^{i\left(kx-\omega t\right)}$$

(11) $$\phi_{3}=\left(De^{-k\xi_{3}z}\right)e^{i\left(kx-\omega t\right)}$$

(12) $$\psi_{3}=\left(Ee^{-k\xi_{s3}z}\right)e^{i\left(kx-\omega t\right)}$$

where $k_{i}^{2}-k^{2}\equiv -k^{2}\xi_{i}^{2}$, $k_{si}^{2}-k^{2}\equiv -k^{2}\xi_{si}^{2}$, and k1=ωωc1c1 is the wavenumber in water, ki=ωωcpicpi,i=2,3 are compressional wavenumbers, and ksi=ωωcsicsi,i=2,3 are shear wavenumbers in the elastic seabed. *A*, $B_{1}$, $B_{2}$, $C_{1}$, $C_{2}$, *D*, and *E* are undetermined coefficients for potentials.

Here, it is assumed that u is the displacement and T is the stress tensor. $u_{ix}$ is the horizontal displacement; $u_{iz}$ is the vertical displacement; $T_{izz}$ is the normal stress; $T_{izx}$ is the shear stress.

The displacements are determined from the velocity potentials as follows:

(13) $$u_{ix}=\frac{1}{-i\omega }\left(\frac{\partial \phi_{i}}{\partial x}-\frac{\partial \psi_{i}}{\partial z}\right),u_{iz}=\frac{1}{-i\omega }\left(\frac{\partial \phi_{i}}{\partial z}+\frac{\partial \psi_{i}}{\partial x}\right)$$

According to the stress–strain constitutive relation, the following equations can be obtained:

(14) $$T_{izz}=\rho_{i}c_{pi}^{2}\left(\frac{\partial u_{ix}}{\partial x}+\frac{\partial u_{iz}}{\partial z}\right)-2\rho_{i}c_{si}^{2}\frac{\partial u_{ix}}{\partial x},T_{izx}=\rho_{i}c_{si}^{2}\left(\frac{\partial u_{ix}}{\partial z}+\frac{\partial u_{iz}}{\partial x}\right)$$

The boundary condition at the interface between water and basalt ($z=H_{1}$) is satisfied by the continuity of the normal displacement, normal stress, and zero tangential stress.

(15) $$T_{1zz}=T_{2zz},u_{1z}=u_{2z},T_{2xz}=0$$

The boundary condition at the interface between basalt and peridotite ($z=H_{2}$) is satisfied by the continuity of displacements and stresses.

(16) $$u_{2z}=u_{3z},u_{2x}=u_{3x},T_{2zz}=T_{3zz},T_{2xz}=T_{3xz}$$

Substituting the general solutions for potentials into the boundary conditions, Equations (15) and (16), a system of homogeneous linear equations can be obtained. Here, we present the equations in matrix form:

(17) $$\left[\begin{array}{ccccccc} a_{11} & a_{12} & a_{13} & a_{14} & a_{15} & 0 & 0\\ a_{21} & a_{22} & a_{23} & a_{24} & a_{25} & 0 & 0\\ 0 & a_{32} & a_{33} & a_{34} & a_{35} & 0 & 0\\ 0 & a_{42} & a_{43} & a_{44} & a_{45} & a_{46} & a_{47}\\ 0 & a_{52} & a_{53} & a_{54} & a_{55} & a_{56} & a_{57}\\ 0 & a_{62} & a_{63} & a_{64} & a_{65} & a_{66} & a_{67}\\ 0 & a_{72} & a_{73} & a_{74} & a_{75} & a_{76} & a_{77} \end{array}\right]\left[\begin{array}{c} A\\ B_{1}\\ B_{2}\\ C_{1}\\ C_{2}\\ D\\ E \end{array}\right]=\left[\begin{array}{c} 0\\ 0\\ 0\\ 0\\ 0\\ 0\\ 0 \end{array}\right]$$

Elements in the first matrix represent the known coefficients in the equations of the boundary conditions. They are a function of *k* such that Equation (17) can be simplified as follows:

(18) $$G\left(k\right)M=O$$

O represents the zero vector. M represents the undetermined coefficients for the potentials, and the values cannot be zero. The non-zero solutions for the coefficients in vector M exist only when the determinant of matrix $G\left(k\right)$ ($\det\left|G\left(k\right)\right|=0$) is zero. The values of *k* that make $\det\left|G\left(k\right)\right|=0$ are therefore the eigenvalues for this question. Once an eigenvalue has been found, the undetermined coefficients in M can be calculated by solving the linear equations. There have been many methods for figuring out the values of *k*, such as the Newton method [27] and bisection [25]. In order to accurately determine the wavenumber *k*, the KRAKENC program [28] is used. Substituting the wavenumber *k* into Equation (17), the undetermined coefficients in M can be calculated by solving the equations. Then, the normal stresses, $T_{zz}$, can be computed by the relations between the stresses and potentials. Acoustic pressure in water is just the negative of $T_{zz}$, according to the definition of acoustic pressure, $p=\frac{1}{i\omega }\rho_{1}{c_{p1}}^{2}\nabla^{2}\phi_{1}$. Eventually, the pressure mode shape function for each *k* can be calculated. This processing of obtaining the pressure mode shape function will be used in subsequent analysis.

The pressure field for a single point source can be represented as a sum of the normal modes. The Scholte wave is treated as the zeroth mode. Then, the pressure can be written as [25]:

(19) $$p\left(r,z\right)=\sum_{m=0}^{\infty }\Phi_{m}\left(r\right)\Psi_{m}\left(z\right)$$

where $\Psi_{m}\left(z\right)$ is a mode shape function, $\Phi_{m}\left(r\right)$ is a mode coefficient, and the subscript *m* is the order of modes. Here, the variable $\Phi_{0}\left(r\right)$ is used to represent the amplitude of the Scholte wave in the acoustic field. We assume that there is no continuous spectrum so that the modes form a complete set. The coefficient $\Phi_{m}\left(r\right)$ can be calculated by applying the operator (Equation (20)) to Equation (19), where $\rho \left(z\right)$ is the media density.

(20) $$\int_{0}^{\infty }\left(\cdot \right)\frac{\Psi_{m}\left(z\right)}{\rho (z)}dz$$

**Figure 2:**
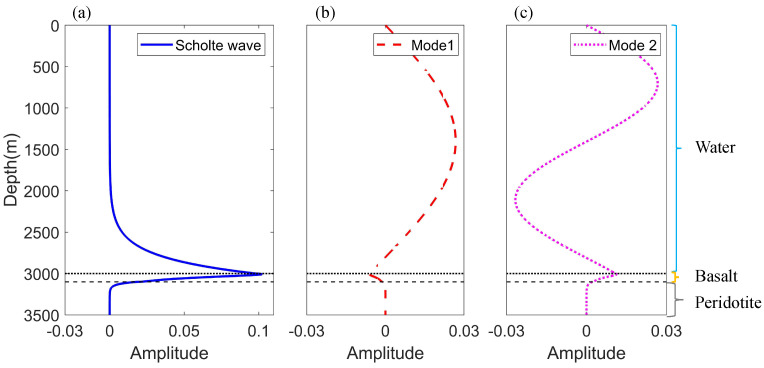
Pressure mode shape functions for a 10 Hz source in the ocean acoustic model. (**a**) Scholte wave. (**b**) Mode 1. (**c**) Mode 2.

Mode analyses based on the elastic normal mode solution are applied to the acoustic field in the model. The pressure mode shape functions $\Psi_{m}\left(z\right)$ at a source frequency of f=10 Hz are illustrated in Figure 2, and the normal stress $T_{zz}$ is shown in the basalt and peridotite layer. The mode shape of the Scholte wave shows that a part of the energy is distributed in the pressure field in seawater. It can be indicated that the excitation intensity of Scholte waves $\Phi_{0}\left(r\right)$ can be affected by source depths and propagation distances.

**Figure 3:**
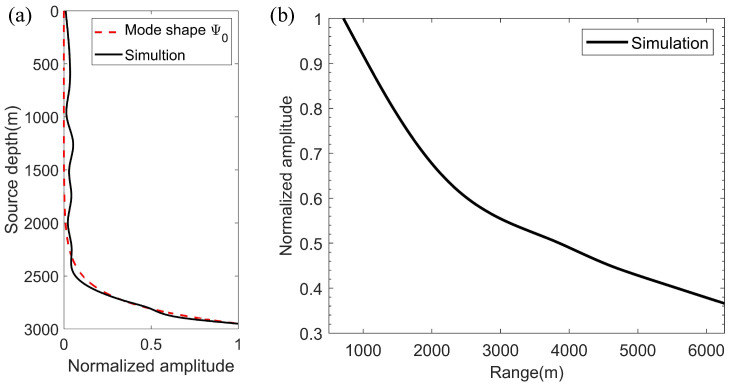
(**a**) Scholte wave amplitude versus source depth. The red dotted line is the mode shape for the Scholte wave and the black line is the simulation result. (**b**) Scholte wave amplitude versus propagation distance.

The finite element method software, COMSOL Multiphysics [29], was applied to simulate the acoustic field in the constructed model. The environmental parameters in the simulation were kept in line with Table 1. The source frequency was f = 10 Hz, and a vertical receiving array was set to obtain pressure in water and normal stress in the bottoms at intervals of 10 m from 0 m to 3500 m. It should be noted that the upper limit of the integral in Equation (20) is infinity, but in this model, it can be found from the mode shape function in Figure 2 that the model distribution at a depth of 3500 m is approximate zero, so it is reasonable to truncate at this depth. Then, the Scholte wave amplitude in the pressure field can be obtained using the mode decomposition method. Figure 3a shows Scholte wave amplitudes, with different source depths, at a 3000-m horizontal distance from the source. The result indicates that the excitation intensity of the Scholte wave increases gradually with the increase of the source depth. It could also explain that Scholte waves can be excited efficiently when the source is at the seafloor or very close to the seafloor. Moreover, Scholte wave amplitudes can vary with range during propagation. When the source depth is SD = 2950 m, the Scholte amplitudes drop monotonically with propagation distance increasing, as shown in Figure 3b.

The simulation results indicate that the amplitude of the Scholte wave increases with an increase in source depths and decreases with an increase in propagation distance. These two characteristics can be applied to detect the Scholte wave, and a water tank experiment is designed according to this principle.

## 3. Tank Experiment

### 3.1. Acoustic Field Analysis for the Laboratory Environment

The acoustic model in Figure 1 is scaled down to laboratory size based on the similarity principle. Due to the close velocities, basalt and peridotite in the seabed are modeled by brass and iron slabs. Table 3 lists a scale model presented at a scale of 1:5000, where the depth and frequency were appropriately modified. Here, the source frequency is 50 kHz, and the water depth is 0.6 m.

**Figure 4:**
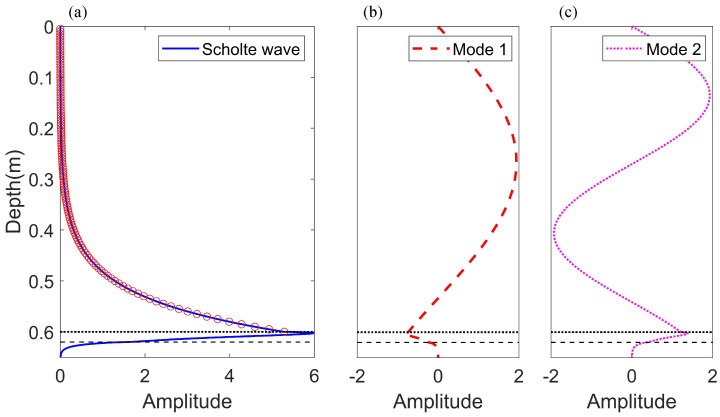
Pressure mode functions for a 50kHz source; the red circles represent the receiver positions. (**a**) Scholte wave. (**b**) Mode 1. (**c**) Mode 2.

Figure 4 shows the pressure mode shape functions $\Psi_{m}\left(r\right)$ of the Scholte wave and normal modes for a 50 kHz source in the water tank. It is illustrated that the mode shape functions in two environments agree well by comparing the results in Figure 2 and Figure 4. Moreover, it reveals that the parameters for the scale model experiment are correct.

### 3.2. Experiment Settings

**Figure 5:**
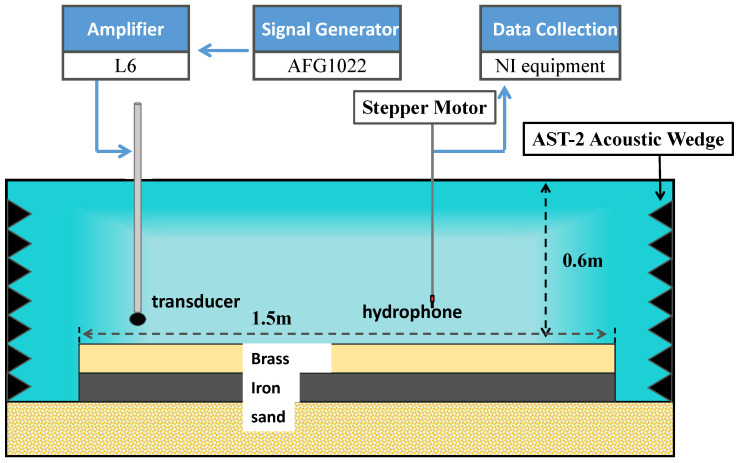
Diagram of the experimental system setup.

The scale model experiment was performed in a water tank (L:3 m × W:2 m × H:1 m). Reflection waves from the tank’s walls are absorbed by absorbing wedges. The tank is filled with water to a height of 0.6 m, where the sound speed in water is measured to be 1485 m/s. A spherical transducer, the source level of which is measured at approximately 140 dB (re 1 µPa.m/V) in the frequency range used in the experiment, is used as the source. An RHCA-7 hydrophone is used as the receiver. The sensitivities of the hydrophones are about −210 dB (re 1 V/µPa) in the frequency range from 20 to 100 kHz. The vertical array, including 119 receiver elements, is obtained by the synthetic aperture method. The source and receiver hydrophones are positioned in water using a robotic apparatus (with an accuracy of 0.01 mm), which allows for accurate positioning. The hydrophone measures depth from 0.5 to 59.5 cm; the depth interval is 0.5 cm. Figure 4 shows the receiver positions in the form of red circles, and the configuration of the experiment equipment is demonstrated in Figure 5.

### 3.3. Experimental Data Analysis

**Figure 6:**
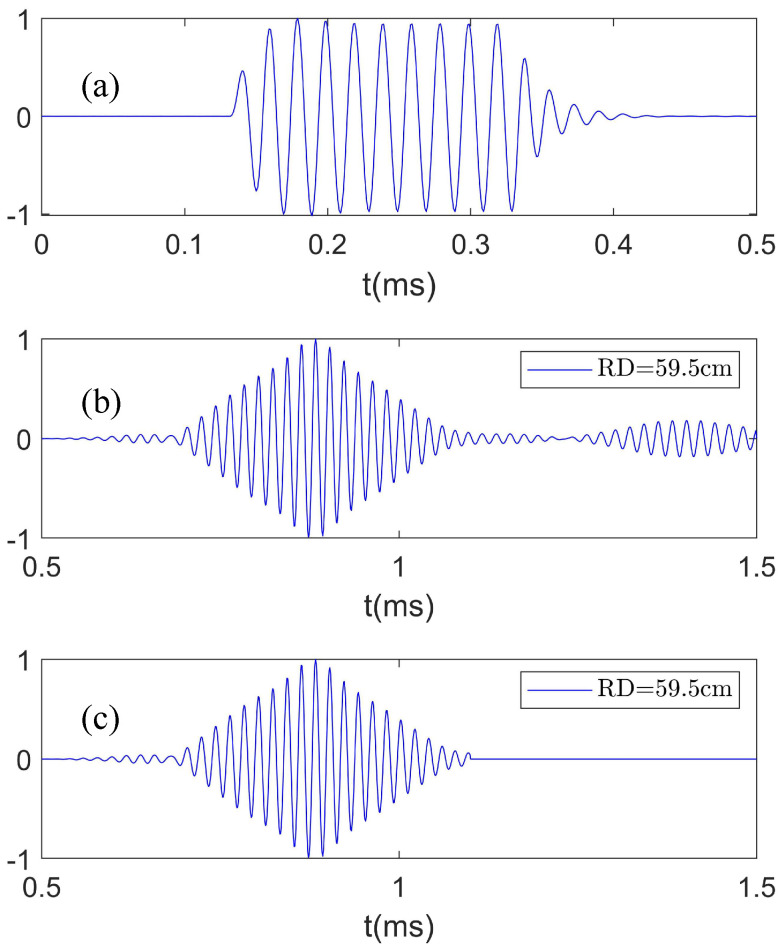
(**a**) Reference signal for a vertical array measurement; (**b**) the received waveform of a hydrophone with a source depth of SD = 55 cm (temporal correlation between the received sound pressure and the reference signal); (**c**) the truncated waveform adopted for identifying Scholte waves.

The source radiates ten cycles of sine waves at a frequency of f = 50 kHz in 1 s time intervals. Figure 6a shows the output signal of a power amplifier, which is used as a reference signal for a measurement with a vertical array of 119 hydrophones. Figure 6b demonstrates the received waveform of a hydrophone, which represents the temporal correlation between the received sound pressure by the hydrophone and the reference signal. By the temporal correlation, the virtual receiving array plays the same role as real receiving arrays of the same length. Therefore, the received waveforms by the virtual array are used to obtain the complex sound pressure required for identifying Scholte waves. To eliminate the effects of reverberation in the water tank, received waveforms are truncated by assuming that the pulse duration of the received signal from the source is almost the same for each hydrophone, and the truncated waveform is shown in Figure 6c.

By the fast Fourier transform of truncated waveforms, we obtain the sound pressure $p\left(r,z\right)$ for the hydrophone at different positions. The sound pressure at 50 kHz is extracted to compose the matrix P, including 119 elements in the depth direction. The mode shape functions in the water tank are demonstrated in Figure 4. Finally, the amplitudes of the Scholte waves can be extracted using the mode decomposition.

**Figure 7:**
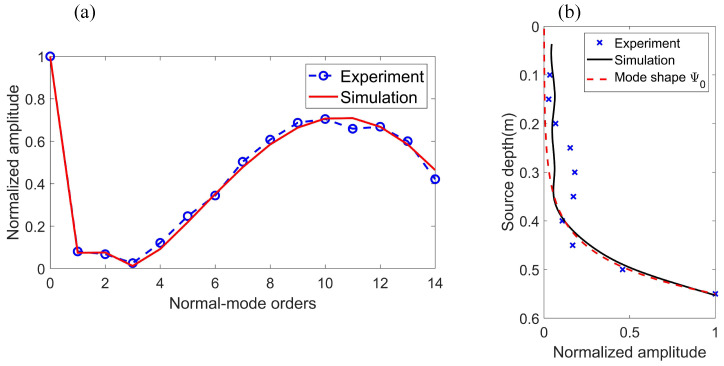
(**a**) Normalized amplitudes of the Scholte wave (0th order) and normal modes for the source depth, SD = 55 cm. The blue circle is the result of the water tank experiment and the red line is the simulation result. (**b**) Normalized amplitudes of the Scholte wave versus source depth. The blue cross is the result of the water tank experiment; the black line is the simulation result; the red dotted line is the mode shape function of the 0th mode.

Ten positions were set evenly for the source in the depth range from 0.1 to 0.55 m; the hydrophone array was at a horizontal distance of 0.6 m away from the source. The result in Figure 7a shows the normalized amplitudes of the Scholte wave and normal modes for a source depth of SD = 55 cm. It was demonstrated that the Scholte wave has the largest amplitude for this source depth, and the amplitudes of each mode in the experiment are in excellent agreement with the simulation result. This result shows that the water tank experiment is feasible and the processing for experimental data is correct. The Scholte wave amplitudes in the experiment field that vary with source depth are presented in Figure 7b. The results reveal that the tendency of the excitation amplitudes of Scholte waves in the experiment is consistent with the theoretical calculations.

**Figure 8:**
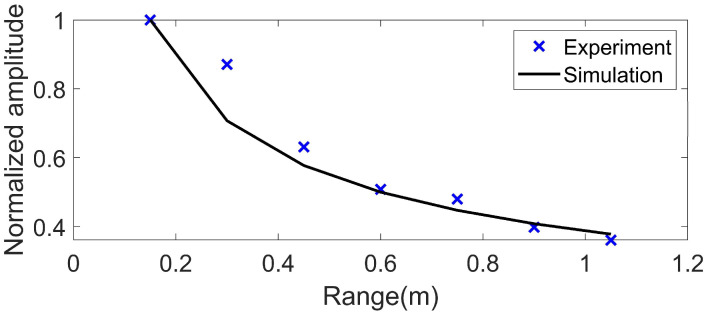
Scholte wave amplitude versus propagation distance. The blue cross is the result of the water tank experiment, and the black line is the simulation result.

Seven positions were set evenly for the receiving array within the horizontal range of 0.15–1.05 m away from the source, and the source was maintained at a depth of SD = 0.55 m. Scholte wave amplitudes in the experiment field that vary with the propagation distance are presented in Figure 8. It is shown that the Scholte wave amplitudes decrease as the propagation distance increases. The tendency of Scholte wave amplitudes in the experiment agrees well with the simulation result. These results show that the detection technique proposed in this paper can detect the Scholte wave at the seafloor successfully.

## 4. Sediment Effect

### 4.1. Experiment Setting

**Figure 9:**
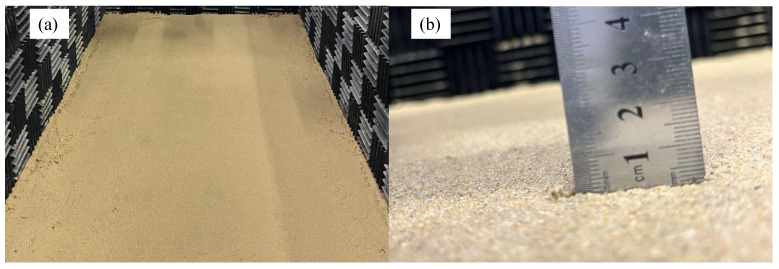
(**a**) The silt sediment layer in the water tank experiment. (**b**) Silt sediment with a thickness of 1 mm.

In practice, there are many types of seafloor sediments. Here, silt was used as the sediment in the water tank experiment. Two cases, a sediment layer thickness of 1 mm and 12 mm, were considered in this research. The corresponding thickness is 5 m and 60 m in the ocean environment according to the scale of 1:5000. The sediment layer in the experiment was very thin, so it was treated as a liquid sediment layer. The model parameters for a 1 mm thickness are listed in Table 4. Here, the thickness of silt is 1 mm. The sediment layer in the water tank experiment and the result of thickness measurement are shown in Figure 9.

The study above proved that the detection technique is valid for detecting Scholte waves at the seafloor. The same technique was applied to the condition with a sediment layer. Mode shapes in this acoustic model can be obtained by the elastic mode solution presented in the theoretical analysis. The process of experimental measurement is similar to the previous experiment, so no more details are provided here.

### 4.2. Experimental Results

**Figure 10:**
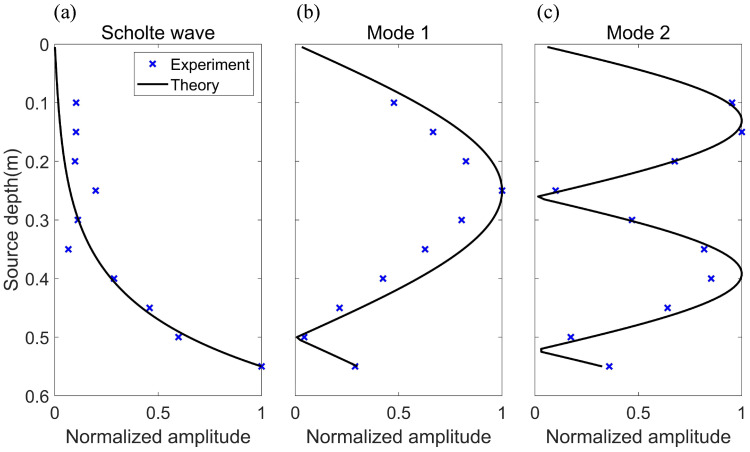
Mode amplitude versus source depth for the Scholte wave and normal modes (thickness of sediment is 1 mm, source frequency f = 50 kHz). The blue cross is the experimental result, and the black line is the theoretical result (mode shape function). (**a**) Scholte wave. (**b**) Mode 1. (**c**) Mode 2.

Figure 10 shows the dependence of the mode amplitude for the Scholte wave and normal modes in the sound field on source depth. The hydrophone array was at a horizontal distance of 0.6 m away from the source. Here, the theory is the mode shape function $\Psi_{m}\left(r\right)$ for each mode. It can be seen from the figure that the results of the experiment and theory agree well with each other. This result indicates that treating the silt as fluid sediment is reasonable, and the Scholte wave can still be excited under this condition.

**Figure 11:**
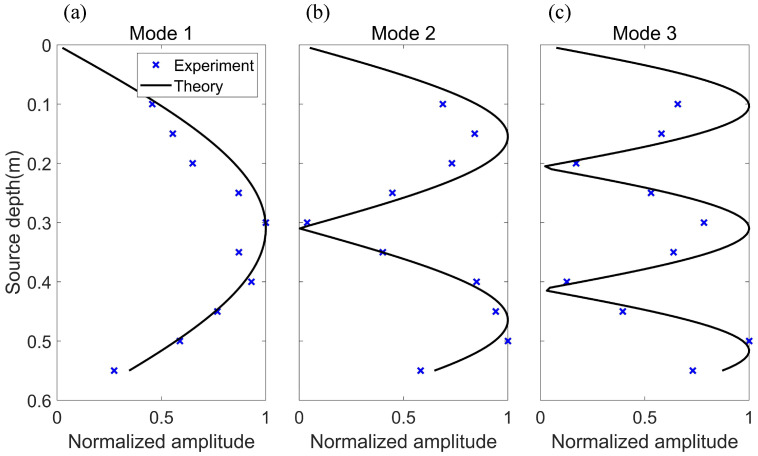
Mode amplitude versus source depth for the Scholte wave and normal modes (thickness of sediment is 12mm, source frequency f = 50 kHz). The blue cross is the experimental result, and the black line is the theoretical result (mode shape function). (**a**) Mode 1. (**b**) Mode 2. (**c**) Mode 3.

The experiment was repeated by changing the thickness of the sediment layer to 12 mm. Figure 11 shows the experimental results for the thickness of the sediment layer being 12 mm. It can be found that no Scholte wave is excited in the sound field under this condition, and the experimental results of normal waves in water are in good agreement with the theory.

The presented results show that the sediment will affect the excitation of Scholte waves. When the sediment layer is thin (the thickness of sediment is 1 mm, and the acoustic wavelength is about 3 cm), the existence of the sediment layer does not affect the excitation of Scholte waves. When the thickness of the sediment layer increases, there is no Scholte wave mode in the acoustic field. Moreover, The results also show that the Scholte wave detection method proposed in this paper is feasible in the sedimentary layer environment.

## 5. Discussion

**Figure 12:**
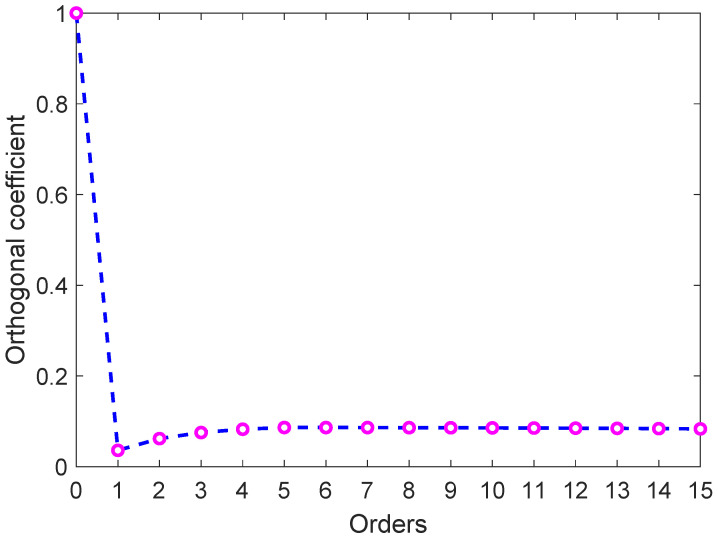
Orthogonal coefficients of the Scholte mode (0th mode) and each mode in water.

Unlike existing methods, this paper proposed a new detection technique based on the acoustic pressure field measurement for identifying Scholte waves according to the excitation and propagation characteristics of Scholte waves. The experimental results show that the detection technique can detect Scholte waves at the seafloor. There are certain discrepancies in the details between the experiments and calculations in this study. Here, the error analysis was performed. Due to the amplitudes of Scholte waves being extracted by mode decomposition in this research, the orthogonality between Scholte and normal modes waves is essential for the results’ accuracy. Theoretically, the upper limit of the integral in Equation (20) is infinity, but only the range of the water depth can be measured in practice, so the modes cannot be strictly orthogonal. Figure 12 shows the orthogonal coefficients of the Scholte mode (0th mode) and each mode in the water column. The Scholte mode is not strictly orthogonal to higher-order normal modes. Therefore, this could be the cause of errors in the amplitudes of Scholte waves.

The acoustic field in this experiment would change when the sediment is added to the model as one layer of the multilayer medium. The normal modes were also changed due to the existence of the sediment layer. This problem still needs further theoretical and experimental research for different seafloor environmental conditions.

## 6. Conclusions

This paper introduces a new detection technique for Scholte waves at the seafloor. According to the actual ocean environment, a series of laboratory experiments with the scaled model of the elastic ocean bottom were designed and performed to detect Scholte waves at the seafloor. The study shows that Scholte wave amplitudes depending on different source depths and propagation distances are in good agreement with the theoretical results. These results indicate that the Scholte wave at the seafloor was detected successfully by the technique.

Furthermore, treating the silt layer in the laboratory experiment as fluid sediment is valid in this study. Some conclusions about the effect of sediment can be reached. When the sediment layer is thin, the existence of the sediment layer does not affect the excitation of Scholte waves. When the thickness of the sediment layer increases, there is no Scholte wave mode in the acoustic field. Moreover, The results also show that the Scholte wave detection method proposed in this paper is feasible in the sedimentary layer environment.

## Figures and Tables

**Table 1:** Parameters of the marine environment.

Media	Layer i	Depth	ρ (g/cm${}^{3}$)	$c_{p}$ (m/s)	$c_{s}$ (m/s)
Seawater	1	3000	1	1500	-
Basalt	2	3100	2.7	5250	2500
Peridotite	3	-	3.28	6500	4000

**Table 2:** Phase velocities of the Scholte wave and normal modes at 10 Hz.

Order	Phase Velocity (m/s)
0	1489.741
1	1500.537
2	1502.139
3	1504.784
4	1508.457

**Table 3:** Parameters of the marine environment.

Media	Layer i	Depth	ρ (g/cm${}^{3}$)	$c_{p}$ (m/s)	$c_{s}$ (m/s)
Water	1	0.6	1	1485	-
Brass	2	0.62	8.54	4640	2050
Iron	3	0.64	7.7	5850	3230

**Table 4:** Media parameters and sizes in the tank experiment.

Media	Layer i	Depth	ρ (g/cm${}^{3}$)	$c_{p}$ (m/s)	$c_{s}$ (m/s)
Seawater	1	0.6	1	1500	-
Silt	2	0.601	1.2	1600	-
Basalt	3	0.621	8.54	4640	2050
Peridotite	4	0.641	7.7	5850	3230

## Data Availability

Not applicable.
